# Use of granulocyte colony‐stimulating factor in the treatment of methimazole‐induced agranulocytosis: a case report

**DOI:** 10.1002/ccr3.1124

**Published:** 2017-09-08

**Authors:** Asha Birmingham, Carissa Mancuso, Craig Williams

**Affiliations:** ^1^ Department of Pharmacy Oregon Health & Science University 3181 S.W. Sam Jackson Park Rd., Mail Code: CR 9‐4 Portland Oregon 97239 USA; ^2^ Department of Pharmacy Memorial Sloan Kettering Cancer Center 1114 1^st^ Ave, 2^nd^ floor New York City New York 10065 USA; ^3^ College of Pharmacy Oregon State University Oregon Health & Science University 2730 SW Moody Ave. Portland Oregon 97201 USA

**Keywords:** Agranulocytosis, antithyroid, colony‐stimulating factor, methimazole, neutropenia

## Abstract

A comparison of this case to previously published reports suggests that granulocyte colony‐stimulating factor may be associated with improved prognosis in severe antithyroid drug‐induced neutropenia, and that weight‐based doses could be an appropriate strategy.

## Introduction

Antithyroid drugs (ATDs) such as methimazole (MMI) and propylthiouracil (PTU) are essential in the conventional management of hyperthyroidism. Current guidelines include strong recommendations for use of these medications in Graves' disease, thyroid storm, hyperthyroidism during pregnancy, and drug‐induced thyrotoxicosis 1. ATD drugs may present an alternative approach for patients who would like to avoid the risks of surgery or radioactive iodine exposure. However, use of ATDs is not without risks. Antithyroid‐induced agranulocytosis (granulocyte count <500/*μ*L) is an uncommon but potentially serious adverse event reported to occur in approximately 0.35% of patients on these agents 2. Although there is evidence to suggest that use of hematopoietic growth factors in drug‐induced agranulocytosis may shorten the duration of neutropenia and reduce infectious or fatal complications, data and clinical guidance for use are limited and inconsistent 3, 4.

## Case Report

A 44‐year‐old woman with a history of hyperthyroidism treated with propranolol was admitted to an outside hospital complaining of ongoing fatigue, headaches, palpitations, and tremor along with sudden onset vertigo and left‐sided weakness. Following discovery of a right cortical vein thrombosis with intraparenchymal hemorrhage and a generalized tonic‐clonic seizure at the referring facility, the patient was transferred to our hospital. Upon admission, she was found to have abdominal pain, temperature of 102°F, heart rate of 121 beats per minute, thyroid‐stimulating hormone (TSH) <0.01 milliunits/L, thyroxine (T4) of 6.7 ng/dL, and a triiodothyronine (T3) of 310 ng/dL, all suggestive of thyroid storm 1. Initial management included a dexamethasone taper for associated adrenal insufficiency, esmolol infusion, and methimazole (MMI) 30 mg by mouth every 6 h. After 9 days, the patients' vital signs and T3 had stabilized and she was discharged on MMI 40 mg daily, propranolol 20 mg twice daily and hydrocortisone 20 mg daily.

Twenty‐one days after discharge (day 30 of MMI treatment), the patient returned to the emergency department (ED) complaining of 1 day of palpitations, fever, nonproductive cough, and sore throat. By the time she presented to the hospital, she was afebrile and her symptoms had resolved. A chest x‐ray was performed showing no acute abnormalities, and blood and urine cultures were negative. Laboratories demonstrated a white blood cell count (WBC) of 4250/*μ*L and an absolute neutrophil count (ANC) of 100/*μ*L, reduced from a baseline of 8700/*μ*L. Differential showed 2.5% neutrophils, 64.9% lymphocytes, 18.8% monocytes, 12% eosinophils, 1.6% basophils, and 0.2% immature granulocytes. Considering the temporal nature of her neutropenia in the setting of recent ATD initiation, MMI‐induced agranulocytosis was suspected (retrospective review indicates a Naranjo algorithm score 7, indicating a “probable” adverse drug reaction) 5. Methimazole was discontinued, and the patient was admitted to the hospital.

On day 2 of admission, her ANC reached a nadir of 80/*μ*L. On day 3, she received 480 mcg (5 mcg/kg) of intravenous (IV) filgrastim once daily with a goal of achieving an ANC of 5000/*μ*L or greater. A rapid response in the patients' ANC was observed, and filgrastim was discontinued after just two doses of 480 mcg (Table 1). The day after the second dose of filgrastim, her ANC reached 11,650/*μ*L and went on to peak at 16,740/*μ*g 2 days after the second dose.

**Table 1:** Medication administration and hematologic laboratory results during initial management

	Normal	Day ‐1	Admission	Day 1	Day 2	Day 3	Day 4	Day 5	Day 6	Day 7
MMI	–	40 mg	40 mg							
Filgrastim IV	–				480 *μ*g	480 *μ*g				
WBC (cells/*μ*L)	4400–11,000	3470	4250	3660	4350	10020	23490	23140	28030	22980
ANC (cells/*μ*L)	1800–7700	90	100	80	160	340	11650	16740	14220	11800
Monocyte (%)	3.5–9.0	11.4	18.8	19.1	19.1	20.3	14.9	12.8	9.2	9.0
Red cell count (million cells/*μ*L)	4.0–5.2	4.79	4.91	4.43	4.57	4.66	4.79	4.56	4.60	4.63
Platelet count (×10^3^/*μ*L)	150–400	379	438	355	395	359	372	339	321	288

ANC, absolute neutrophil count; IV, intravenous; MMI, methimazole; WBC, white blood cells.

The patients' ANC remained within or above the normal range for the duration of her admission. No fevers or infectious complications were documented. The patient reported bone pain during filgrastim administration which is relatively common and was successfully treated with oxycodone and loratadine. Mild flu‐like symptoms are also fairly common but did not occur in this patient. Several days after administration, the patient also reported unusual dreams thought to be associated with high‐dose steroids; no other adverse effects were noted. The patients' hyperthyroidism was ultimately managed with a total thyroidectomy and thyroid replacement therapy. After 17 days, the patient was discharged with a T4 of 1.2 ng/dL and ANC normalized to 5250/*μ*L. In the 6 months following discharge, her hematologic counts remained within normal limits. Aside from one visit to an outside ED for recurrent bone pain 2 months after discharge, no complaints specific to management of her agranulocytosis were documented.

## Discussion

Antithyroid drugs work by decreasing synthesis of thyroid hormone via inhibition of thyroid peroxidase 2. Evidence demonstrating effects on immunologic pathways also suggest an additional mechanism for both the efficacy and adverse effects of ATDs.

### Mechanism of agranulocytosis

Agranulocytosis usually occurs within the first 90 days of ATD treatment, though presentation has been reported up to a year or more after initiation and even after restarting a previously well‐tolerated ATD 2, 4, 6. The incidence of MMI‐induced agranulocytosis appears to be dose‐related. However, cross sensitivity between PTU and MMI exists, so dose reductions, rechallenging, or alternative ATD therapy should be avoided after the occurrence of agranulocytosis 2, 4.

The mechanism of ATD‐induced agranulocytosis is believed to be immune‐mediated 2, 4. Studies among patients with PTU‐induced neutropenia have demonstrated the presence of granulocyte‐specific IgG antibodies 7, as well as compliment‐mediated cytotoxicity via interaction of antineutrophil cytoplasmic antibodies and their neutrophil surface‐bound targets (proteinase 3 and myeloperoxidase) 8. Toxicity may result from a combination of drug‐dependent, hapten‐type and autoantibody‐type mechanisms directed at mature granulocytes as well as immature, hematopoietic progenitor cells 9. Genetic factors may also predispose patients to ATD‐induced agranulocytosis, with human leukocyte antigen (HLA) allele DRB1*08032 being associated with increased risk among Japanese patients 4.

### Duration of agranulocytosis and the effect of granulocyte colony‐stimulating factor

The duration of drug‐induced agranulocytosis has been reported to range from 4 to 56 days, and longer recovery times are often associated with hypoplastic bone marrow 10. Granulocyte colony‐stimulating factor (G‐CSF) is a well‐established treatment for reducing the duration of chemotherapy‐induced neutropenia, and previous experience suggests that it may also have a role in the treatment of adverse events associated with nonchemotherapy medications. In one systematic review of drug‐induced agranulocytosis (including 87 MMI‐, PTU‐, or carbimazole‐related cases), administration of hematopoietic growth factors was associated with a shorter duration of neutropenia compared to no treatment (8 days vs. 9 days, *P* = 0.015, and 7.5 days vs. 10 days in asymptomatic patients, *P* = 0.007) 3. Use of G‐CSF or granulocyte macrophage colony‐stimulating factor (GM‐CSF) was also associated with fewer infectious or fatal complications (14% vs. 29%, *P* = 0.03), suggesting that hematopoietic growth factors may be an effective treatment for drug‐induced neutropenia.

When analyzing the effects of hematopoietic growth factors on ATD‐induced agranulocytosis, specifically, results are less clear. Some case reports and one prospective, randomized controlled trial suggested a lack of effect in reducing the duration of ATD‐induced neutropenia using G‐CSF, though the low doses used (75–250 *μ*g subcutaneously once daily) have been suggested to have contributed to this lack of effect 11, 12, 13. One retrospective analysis comparing 20 patients with ATD‐induced agranulocytosis found a significantly shorter duration of neutropenia (6.8 ± 4 vs. 11.6 ± 5 days, *P* = 0.046), as well as shorter durations of antibiotic treatment and hospital length of stay in patients treated with 300 *μ*g/day compared to those without growth factor treatment 14. Currently, many experts recommend use of G‐CSF in ATD‐induced agranulocytosis, and some suggest preferential use among patients with poor prognosis (including the presence of renal failure, cardiac or respiratory failure, systemic autoinflammatory disease, severe infection, age >65 years, or ANC <100 cells/*μ*L) 2, 13, 14, 15.

In some patients with severe neutropenia (ANC less than 100 cells/*μ*L), time to recovery after discontinuation of the implicated ATD and G‐CSF administration has ranged from roughly 6 to 14 days using G‐CSF doses ranging from 75 to 375 *μ*g/day 12, 16, 17, 18. In two of the patients included in these reports, ANC recovered after their G‐CSF dose was increased from 75 to 300 *μ*g/day, though the relevance of the association between increased dose and recovery is unknown 16. Successful treatment of ATD‐related cases with higher G‐CSF doses has also been reported. In a case series including four patients with thiamazole‐induced agranulocytosis, twice daily 300 *μ*g subcutaneous G‐CSF was associated with rapid improvement of neutropenia 10. Within 2–4 days, ANC increased from 0/*μ*L to >6000/*μ*L in three patients and from 360/*μ*L to >6000/*μ*L in one patient. Despite the high‐dose of hematopoietic growth factor, no adverse effects were reported and authors concluded that high doses of G‐CSF in the setting of potentially life‐threatening neutropenia may be reasonable.

### Characteristics of patients with long durations of neutropenia

Severe neutropenia or pronounced suppression of myeloid precursors has been associated with a prolonged recovery time and failure to respond to G‐CSF 12, 19. In an observational study including 12 subjects treated with 75 *μ*g/day of G‐CSF, patients with bone marrow granulocyte to erythrocyte count ratios (G:E) <0.5 (normal ≥2.5) or peripheral granulocyte counts <100 cells/*μ*L had ANC recovery times that were significantly longer compared to those with a G:E greater than or equal to 0.5 (2.2 ± 0.6 days vs. 9.8 ± 1.3 days, *P* < 0.001) or an ANC >100 cells/*μ*L (2.2 ± 0.4 vs. 8.6 ± 1.3 days, *P* < 0.001) 20. Thus, a greater risk of poor prognosis may be associated with severe neutropenia or pronounced suppression of myeloid progenitors, and bone biopsy upon presentation can help identify patients requiring more aggressive management strategies.

In another observational study of 54 patients with Graves' disease, investigators performed serial measurements of serum G‐CSF levels among six patients with MMI‐induced agranulocytosis 21. At the onset of agranulocytosis, bone marrow specimens were obtained, and treatment with G‐CSF 100 *μ*g/day was initiated until an ANC >1000 cells/*μ*L was achieved. It was observed that patients with greater suppression of immature granulocyte cell types reached higher concentrations of endogenous G‐CSF, correlating with longer recovery times (*r* = 0.824, *P* < 0.05). Authors speculated that patients with greater bone marrow suppression may have required higher doses of G‐CSF in order to reach peak concentrations equivalent to the observed endogenous peaks and to see the benefit of shortened recovery time.

### Patient Case

Relative to previously published case series, a couple points are worth noting in our case. First, we used G‐CSF at a fairly aggressive dose and via the IV route. While subcutaneous dosing of G‐CSF has been found to generally be superior, IV use remains a viable clinical option 22.

Next, our patients' recovery of ANC was rapid despite a severe neutropenia. While it is possible that G‐CSF merely augmented a recovery that was already underway, it should be noted that the patients' ANC dropped further while on our clinical service. Thus, the myelosuppressive effects of ATD therapy were still acute when G‐CSF therapy was begun.

Last, in the context of that rapid recovery, it is likely that our patient suffered a reduction in granulopoiesis without a significant inhibition of granulocytic precursors, as suggested by her monocytosis. A shorter duration of neutropenia may be expected in such a case. However, severe agranulocytosis, as seen in this patient, is not typically associated with such a rapid and sustained recovery in ANC. Considering that some degree of dose response has been supported by previous literature, the higher weight‐based dose used in this case almost certainly contributed to the short duration of neutropenia and the favorable outcome which was observed.

## Conclusion

All patients receiving ATDs should be counseled to seek immediate medical attention for signs of infection, including fever and sore throat. For such patients who present with neutropenia, the offending agent should be promptly discontinued, and all ATDs subsequently avoided. Use of hematopoietic growth factors to shorten the duration of neutropenia should also be considered, especially for patients with characteristics suggesting poor prognosis. The heterogeneity of existing studies and case reports complicates the determination of best G‐CSF dosing strategy in ATD‐induced agranulocytosis, however. In our patient, a once daily IV dose of filgrastim 480 *μ*g IV (5 *μ*g/kg) was associated with a rapid improvement in ANC, increasing from 80/*μ*L to 11,650/*μ*L after only 2 days of treatment.

In summary, the case presented here adds additional support to the literature for a 5 *μ*g/kg dose of IV G‐CSF and suggests that such dosing can be associated with a rapid recovery from neutropenia after ATD‐induced agranulocytosis.

## Authorship

ARB: contributed to the literature search and was the primary author. CM: contributed to the literature search and preliminary summary, content revision. CW: contributed to article conception, content revisions and supporting materials.

## Conflicts of Interest

The authors of this presentation have nothing to disclose concerning possible financial or personal relationships with commercial entities that may have a direct or indirect interest in the subject matter of this document. All coauthors are aware of content presented here. This case study has been deemed exempt by the Institutional Review Board.
